# Comparison of anti-inflammatory activities of an anthocyanin-rich fraction from Portuguese blueberries (*Vaccinium corymbosum* L.) and 5-aminosalicylic acid in a TNBS-induced colitis rat model

**DOI:** 10.1371/journal.pone.0174116

**Published:** 2017-03-22

**Authors:** Sónia R. Pereira, Rita Pereira, Isabel Figueiredo, Victor Freitas, Teresa C. P. Dinis, Leonor M. Almeida

**Affiliations:** 1 CNC-Center for Neuroscience and Cell Biology, Coimbra, Portugal; 2 Faculty of Pharmacy, University of Coimbra, Coimbra, Portugal; 3 Department of Chemistry and Biochemistry, Faculty of Sciences, University of Porto, Porto, Portugal; Cairo University Faculty of Pharmacy, EGYPT

## Abstract

Despite the actual therapeutic approaches for inflammatory bowel disease (IBD), efficient and secure alternative options remain a research focus. In this context, anthocyanins seem promising natural anti-inflammatory agents, but their action mechanisms and efficacy as compared with established drugs still require more clarification. The main aim of this study was to compare the anti-inflammatory action of a chemically characterized anthocyanin-rich fraction (ARF), obtained from Portuguese blueberries (*Vaccinium corymbosum* L.), with that of 5-aminosalicylic acid (5-ASA), a first-line drug in IBD, in a 2,4,6-trinitrobenzenesulfonic acid (TNBS)-induced colitis rat model. Such fraction showed a high content and great molecular diversity of anthocyanins, with malvidin-3-galactoside and petunidin-3-arabinoside in the highest concentrations. After daily administration by intragastric infusion for 8 days, ARF, at a molar anthocyanin concentration about 30 times lower than 5-ASA, showed a higher effectiveness in counteracting the intestinal inflammation, as assessed by i) body weight variation and colon damage score, ii) reduction in leukocyte infiltration, iii) increase in antioxidant defenses and iv) by downregulation of inducible nitric oxide synthase (iNOS) and cyclooxygenase-2 (COX-2) in colon tissue homogenates. The strong inhibition of COX-2 expression seems to be a crucial anti-inflammatory mechanism common to both ARF and 5-ASA, but the additional higher abilities of anthocyanins to downregulate iNOS and to decrease leukocytes infiltration and to increase antioxidant defenses in colon may account for the much higher anti-inflammatory action of anthocyanins. These data may contribute to the development of a promising natural approach in IBD management.

## Introduction

Inflammatory bowel disease (IBD) is a chronic and relapsing disorder of the gastrointestinal tract (GI), associated with an exacerbated intestinal immune response to an innocuous stimulus [1], leading to an upregulation of pro-inflammatory mediators which may trigger the onset and perpetuation of the disease [2].

Although several therapeutic strategies have been proposed [3], they are not free of adverse effects and so the development of new effective and safer ones remains a focus of interest. In this context, the use of natural products and dietary components has been gaining popularity for the treatment of inflammatory disorders [4–6], but scientific evidences regarding their efficacy and action mechanisms are still required.

Blueberries are among the fruits with potential health benefits associated with their high content in anthocyanins, a group of flavonoids widespread in human diet [7], which have been reported as potential therapeutic agents for several inflammatory diseases, including IBD [8,9]. Anthocyanins have been largely recognized by their potent antioxidant properties and ability to modulate crucial signalling pathways and gene regulation of several inflammatory enzymes and cytokines [5,9–13]. Therefore, beyond these modulatory roles, their antioxidant activity related to the capacity to scavenge reactive oxygen and nitrogen species or to activate cellular endogenous antioxidant systems [14,15], may be of major importance in countering the oxidative stress in IBD [16].

Despite the controversy regarding the bioavailability of anthocyanins [17,18], they can reach concentrations up to several hundred micromolar in the gastrointestinal tract [19], due to their abundance in diet and poor intestinal absorption. Recent studies have already shown that oral consumption of berry anthocyanins attenuates inflammation in mice models of colitis [20], but more information is required concerning their anti-inflammatory action mechanisms and efficacy as compared with standard anti-inflammatory drugs used in IBD patients.

Thus, in this context, we have already demonstrated, in an *in vitro* intestinal cell model, the higher anti-inflammatory activity of cyanidin-3-glucoside in comparison with 5-aminosalicylic acid (5-ASA) [13], a well-established anti-inflammatory drug in IBD. Now, in this work, we aimed to investigate the anti-inflammatory action of an anthocyanin-rich fraction (ARF) of Portuguese blueberries (*Vaccinium corymbosum* L.), in a TNBS-induced colitis rat model, in comparison with 5-ASA.

The efficacy of the treatments was firstly evaluated by body weight variations and extent of colonic mucosal injury. To gain a better insight into the anti-inflammatory mechanisms involved, we assessed in the colon tissue, i) the active myeloperoxidase (MPO) and the alkaline phosphatase (ALP) activity, as markers of inflammation, ii) the intracellular redox status, through evaluation of glutathione (GSH)/glutathione disulphide (GSSG) ratio and glutathione peroxidase (GPX) activity, and iii) the expression of the pro-inflammatory enzymes, inducible nitric oxide synthase (iNOS) and cyclooxygenase-2 (COX-2).

## Materials and methods

### Reagents

Laboratory chemicals, namely 5-aminosalicylic acid, 2,4,6-trinitrobenzenesulfonic acid solution (TNBS), 5,5′-dithiobis(2-nitrobenzoic acid) (DTNB), glutathione reductase, L-glutathione reduced (GSH) and oxidized (GSSG) forms, β-NADPH, 5-sulfosalicylic acid, 2-vinylpyridine, 2,3-diaminonaphthalene (DAN), o-dianisidine dihydrochloride, hexadecyltrimethylammonium bromide (CTAB), protease inhibitor cocktail and general laboratory chemicals were purchased from Sigma-Aldrich (St Louis, Missouri, USA). Microcon-10kDa centrifugal filters were obtained from Merck Millipore (Merck KGaA, Darmstadt, Germany).

Rabbit polyclonal antibody to iNOS was purchased from Cell Signalling Technology (Leiden, The Netherlands); rabbit polyclonal antibody to COX-2 was purchased from Abcam (Cambridge, UK); goat polyclonal antibody to MPO was purchased from Santa Cruz Biotechnology (Santa Cruz, CA, USA); mouse monoclonal antibody to β-actin was purchased from Sigma-Aldrich (St Louis, Missouri, USA); anti-rabbit, anti-mouse and anti-goat IgG secondary antibodies were purchased from Abcam (Cambridge, UK).

### Blueberry samples

The blueberries (*Vaccinium corymbosum* L, cultivar Bluecrop) were collected at the time of peak production, from the central region of Portugal. They were obtained from biological agriculture (Biogrêsso, Portugal) and kept frozen at -80°C until use.

### Preparation of anthocyanin-rich fraction

The anthocyanin-rich fraction was prepared according to Oszmianski, et al [21], as modified by Youdim (2002) [22]. Briefly, a total extract was obtained by homogenization of 30 g of the frozen fruits in 150 ml of methanol, acetone, water and formic acid mixture (40:40:20:0.1 v/v/v/v). Then, the extract was centrifuged at 2000*g* for 10 min and the supernatant was dried by rotatory evaporation at 40°C under vacuum. The resulting residue was dissolved in deionized water and applied to an activated Sep-Pak C18 column (Waters Corporation, Milford, Massachusetts, USA). The column was then washed with 2 volumes of acidified water (0.01% HCl) to remove sugars and phenolic acids, and 2 volumes of ethyl acetate to elute other phenolic compounds. The anthocyanins were eluted only in the presence of acidified methanol (0.01% HCl) [23]. This fraction was then dried at low pressure, and further resolubilized in a 0.9% NaCl solution. The anthocyanin-rich extract was kept at -80°C, under nitrogen, until use and will be further referred here as ARF.

### Total phenols and total anthocyanins assays

The total content in phenolic compounds was assessed spectrophotometrically by the Folin-Ciocalteau reagent, as described by George et al [24]. Results were expressed as grams of gallic acid equivalents (GAE) per volume of ARF (L) and as milligrams of GAE per 100 g of blueberries.

Total monomeric anthocyanins in the ARF were estimated by a pH differential method described by Giusti et al [25], by using two buffer systems. The fraction was previously diluted at a ratio of 1:800 (v:v) either with 0.025 M KCl (pH 1) or with 0.4 M sodium acetate buffer (pH 4.5) and after an equilibration period (15 min), the absorbance spectrum of each solution was recorded between 400 and 700 nm in a Perkin Elmer Lambda 45 spectrophotometer. The anthocyanin content was calculated in terms of malvidin-3-glucoside (malv3glc) equivalent, using the maximum of absorbance measured around 530 nm, the molar absorptivity of 20200 and the molecular weight of 493.4. The results were expressed as grams of malv3glc equivalents/L of ARF and as milligrams of malv3glc equivalents/100 g blueberries.

### HPLC-DAD

The extract was analysed by HPLC with direct injection of the samples (20 μL) (Hitachi L-2130) in a reverse phase C18 column with 250 x 4.6 mm i.d. (Merck KGaA, Darmstadt, Germany); the detection was performed at 511 nm, using a diode array detector (DAD) (Hitachi L-2455). The solvents used (mobile phase) were A: H_2_O/HCOOH (9:1), and B: H_2_O/CH_3_CN/HCOOH (6:3:1), in a gradient of 20–85% from solution B for 70 min at a flow rate of 1.0 mL/min. The column was then washed with 100% of solution B for 20 min. Before each use, the column was stabilized for the initial conditions for another 20 min [26].

### Animals and experimental design

Four-week-old male Wistar rats were obtained from Charles River Laboratories (Barcelona, Spain), and maintained under standard conditions (temperature 24–25°C, humidity 70–75%, lighting regimen of 12 h light/dark cycle), in plastic cages with free access to water and pellet food. Animal welfare and experimental procedures were carried out in accordance to the European Union regarding animal experimentation (Directive of the European Counsel 86/609/EEC) and approved by the Portuguese National Authority for Animal Health (Direcção-Geral de Alimentação e Veterinária—DGAV). All efforts were made to minimize the animals suffering and to reduce the number of animals used. An early humane endpoint criteria was adopted: although weight loss was expected in this disease model, if this loss exceeded 20% of the initial weight, animals would be euthanized. The rats were randomly divided into four groups: (i) non-colitic control (*n* = 10); (ii) TNBS-colitic control (*n* = 10); (iii) TNBS-induced rats treated with ARF 10 mg.kg^-1^ (*n* = 10); and (iv) TNBS-induced rats treated with 5-ASA 100 mg.kg^-1^ (*n* = 10). The ARF and the therapeutic agent were administered solubilized in a mixture of 0.9% NaCl and 0.5% carboxymethylcellulose (vehicle), in a single dose by oral gavage, during 8 days after colitis induction by TNBS.

The dose of ARF used was chosen on basis of preliminary experiments, as the lowest to obtain safety and efficacy in the referred colitis rat model, and that of 5-ASA was as reported in literature [5,27] and taking into consideration the dose translation from animal to humans [28].

### Induction of colitis

Colonic inflammation was induced as described by Siddiqui et al [29]. Rats were fasted overnight and were then anesthetized with ketamine (50 mg.kg^-1^) and chlorpromazine (2.3 mg.kg^-1^), to allow the rectal administration of TNBS. Briefly, the tip of a polyethylene catheter was advanced transanally 8 cm into the distal colon and a single dose of TNBS was instilled intraluminally (10 mg of TNBS dissolved in 0.25 mL of 50% ethanol) to induce colitis. The animals were maintained in a head-down position for approximately 60 seconds to prevent leakage of the infusate. Non-colitic control group was subjected to the same procedure, infusing a saline buffer instead of TNBS. After one day, the rats were treated for 8 days as assigned previously.

### Macroscopic assessment and scoring of TNBS colitis severity

Rats were sacrificed by cervical dislocation under anaesthesia with ketamine (50 mg.kg^-1^) and chlorpromazine (2.3 mg.kg^-1^). The colon was excised, opened longitudinally and washed in saline solution. Afterwards, the colon segment was weighed and its length measured. Each colon was scored for macroscopically visible damage by two observers unaware of the treatment, according to the criteria reported by Bell et al [30] and described in (**Table 1**). Representative whole gut specimens were taken from the inflamed colon region, or its equivalent segment in the control group, and stored at -80°C for subsequent biochemical measurements.

**Table 1:** Criteria for the colonic macroscopic damage score assessment.

Score	Criteria
0	No damage
1	Hyperemia, no ulcers
2	Linear ulcer with no significant inflammation
3	Linear ulcer with inflammation at one site
4	≥2 sites of ulceration = inflammation
5	≥2 major sites of ulceration and inflammation or one site of ulceration/inflammation extending >1cm along the length of the colon
6	If damage covers >2cm along the length of the colon, the score is increased by 1 for each additional centimeter of involvement

Criteria according to Bell et al [30]

### Myeloperoxidase activity

Colonic mucosa was assayed for MPO activity as described by Stucchi et al [31], with slight modifications. Samples excised from each animal were thawed, and approximately 30–50 mg of mucosa was homogenized on ice with a Polytron tissue homogenizer in 4 ml of ice-cold 5 mM phosphate buffer (pH 6.0). To remove haemoglobin and other blood products that could interfere with this assay, the homogenate was centrifuged at 30000*g*, for 30 min, at 4°C and the supernatants discarded and the pellet resuspended in phosphate buffer, for three times. Finally, the pellet was solubilized in 10 volumes of ice-cold hexadecyltrimethylammonium bromide (0.5%) in phosphate buffer (50 mM, pH 6.0) and subjected to three cycles of freezing/thawing to extract the enzyme. The extract was allowed to stand at 4°C for 20 min and then centrifuged at 12500*g* for 15 min at 4°C. MPO activity was evaluated in the supernatant, by mixing 10 μL of this supernatant with 2.99 mL of 50 mM phosphate buffer, pH 6.0, containing 0.167 mg.mL^-1^ of *o*-dianisidine dihydrochloride and 0.0005% of H_2_O_2_. The absorbance decrease of the reaction mixture was monitored at 460 nm for 2 min. Results were expressed as U MPO.mg^-1^ tissue, being one unit of MPO activity defined as the amount of enzyme that degrades 1 μmol of hydrogen peroxide per minute.

### Alkaline phosphatase activity

ALP activity was measured in colon tissue samples by the method described by Bessey et al. [32] as modified by Sanchez et al. [33]. The samples were homogenized in cold saline solution (1:20 w/v) and centrifuged at 7000*g* for 10 min at 4°C. The supernatants were used to determine spectrophotometrically the mucosal ALP activity, by mixing 100 μL of each sample with 1 mL of glycine (50 mM, pH 10.4), containing 5.5 mM *p*-nitrophenylphosphate and 0.5 mM magnesium chloride. After incubation for 30 min at 37°C, 10 mL of 0.02 M sodium hydroxide were added to stop the enzymatic reaction, and the *p*-nitrophenol formed was measured at 405 nm. The protein content was determined by the Bradford reaction using the Bio-Rad protein assay dye reagent (Bio-Rad, Hercules, California, USA), to express ALP activity as mU. g^-1^ of protein. One unit of ALP activity is defined as the amount of enzyme that catalyzes the transformation of one micromole of substrate per minute.

### Measurement of GSH/GSSG ratio

Total glutathione and GSSG were quantified in colon samples by an enzymatic recycling assay, essentially as described by Griffith et al [34]. The colon samples were previously treated by homogenization in extraction buffer containing 0.1% Triton X-100, 0.6% sulfosalicylic acid, 0.1 M KH_2_PO_4_ and 5 mM Na_2_PO_4_, pH 7.5), followed by centrifugation at 3000*g*, at 4°C, for 10 min [35]. Total glutathione (GSH + GSSG) was assessed in the supernatant in a 96-well plate. Briefly, 20 μL of supernatant was added to 120 μL of freshly prepared 5 mM DTNB plus glutathione reductase (1:1 v/v) in 0.1 M potassium phosphate buffer and 60 μL of 2.4 mM β-NADPH, also prepared in the same phosphate buffer. The absorbance due to the yellow dianion formed by the reaction of thiol groups and DTNB, was immediately read at 412 nm, using a Synergy HT plate reader (Bio-Tek Instruments) and followed for 2 min, with reading intervals of 30 sec. For the GSSG determination, the procedure was similar but prior to the assay, the GSH was blocked by derivatization using 2-vinilpyridine, by incubating 100 μL of the tissue samples with 2 μL of this masking agent and 6 μL of triethanolamine, during 1 h at room temperature, in a fume hood. Colon glutathione content (total and oxidized) was calculated using concurrently run standard curves in nmol per mg of cellular protein, and expressed as GSH/GSSG ratio.

### Measurement of glutathione peroxidase activity

The activity of GPX in colon samples was evaluated using a colorimetric assay kit purchased from Abcam (Cambridge, UK), which monitors GSH oxidation by recording the consumption of NADPH at 340 nm. Colonic tissue extraction and enzymatic activity were performed in accordance to the manufacturer’s protocols and the results expressed in U per gram of colonic tissue.

### Western-blot analysis of MPO, COX-2 and iNOS expressions

Colonic tissues were processed according to the method described by Sanchez-Fidalgo et al. [36] slightly modified. Briefly, frozen colonic tissues were weighed and homogenized in ice-cold lysis buffer (50 mM Tris–HCl, pH 8; 150 mM NaCl; 0.1% Triton X-100; 0.1% protease inhibitor cocktail; 1 mM PMSF; 0.5 mM EDTA; and 8 mM MgCl_2_). Homogenates were incubated at 4°C for 2 h with stirring, and then cell debris were removed by centrifugation (12000*g*, 20 min, at 4°C) and the supernatants (cytoplasmic extracts) were collected and stored at -80°C until use. Protein concentration was determined using the Bio-Rad protein assay kit. Aliquots of the supernatant, containing equal amount of reduced and denaturated proteins (80 μg), were separated by SDS/PAGE electrophoresis in a 10% (v/v) acrylamide gel and further transferred onto polyvinylidene difluoride (PVDF) membranes (Amersham Biosciences, UK) by electroblotting, using the Trans-Blot Turbo Transfer System of Bio-Rad (Hercules, California, USA). The membranes were blocked with skimmed milk in TBS-T buffer (20 mM Tris-HCl, 150 mM NaCl, 0.1% Tween) to avoid non-specific binding. Afterwards the membranes were probed overnight, at 4°C, with specific primary antibodies against MPO, COX-2, iNOS and β-actin. Each membrane was then washed three times with TBS-T and incubated with the respective alkaline phosphatase-conjugated secondary antibodies, for 1 h, at room temperature. Immunodetection was performed by chemifluorescence after blots exposition to enhanced chemifluorescent reagent in a Typhoon 9000 scanner (Amersham Biosciences, UK) and analysed with the ImageQuant^TM^ software from Amersham Biosciences (UK). β-Actin was used as control for protein loading.

### Statistical analysis

All results are expressed as the mean ± S.E.M. of at least 8 animals per group, and each sample assayed in duplicate. Differences between means were tested for statistical significance using a one-way or two-way analysis of variance (ANOVA), using Dunnett’s multiple comparisons test as post hoc test. All statistical analysis was carried out with the GraphPad Prism version 5 (GraphPad Software, Inc., CA, USA) and values of *P* < 0.05 were accepted as statistically significant.

## Results

### The blueberry extract showed a high content and a great diversity of anthocyanins

**Fig 1:**
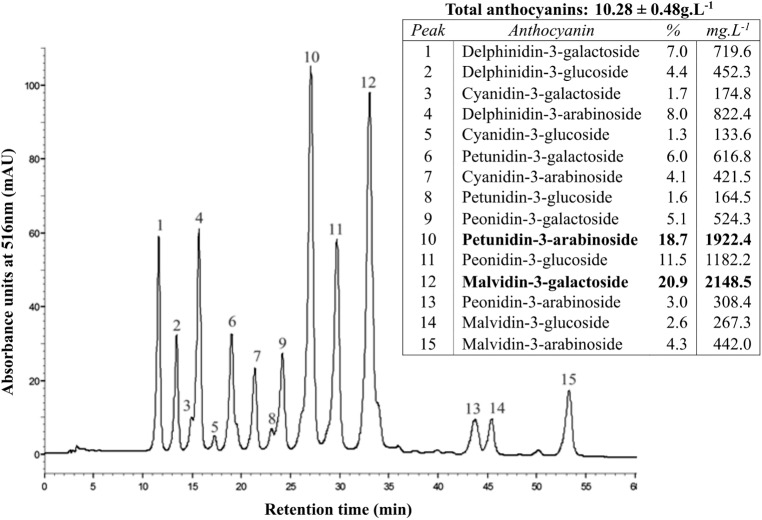
HPLC-DAD chromatogram of blueberry anthocyanin-rich fraction. The peak identification and the contents of the different anthocyanins are indicated in the inset table.

Anthocyanin analysis of the anthocyanin-rich fraction showed a high content of anthocyanins, about 10.28 ± 0.48 g/L, or 98.75 ± 5.71 mg/100g of fruit, in terms of malvidin-3-glucoside. The total phenolic content of this fraction was 12.92 ± 1.59 g/L, or 119.96 ± 14.80 mg/100g of fruit, in terms of gallic acid equivalents. Moreover, using HPLC-DAD we could confirm that this fraction showed also a wide variety of anthocyanin molecules (15 peaks detected) as shown in **Fig 1**. The anthocyanins detected were, in descending order of amount, malvidin, petunidin, peonidin, delphinidin and cyanidin, conjugated with either galactose, glucose or arabinose, being malvidin-3-galactoside (20.93%) and petunidin-3-arabinoside (18.70%) the main anthocyanins detected (**Fig 1 inset**).

### The anthocyanin-rich fraction improved more efficiently than 5-ASA the recovery of body weight and the macroscopic colonic damage in TNBS-induced colitis in rats

**Fig 2:**
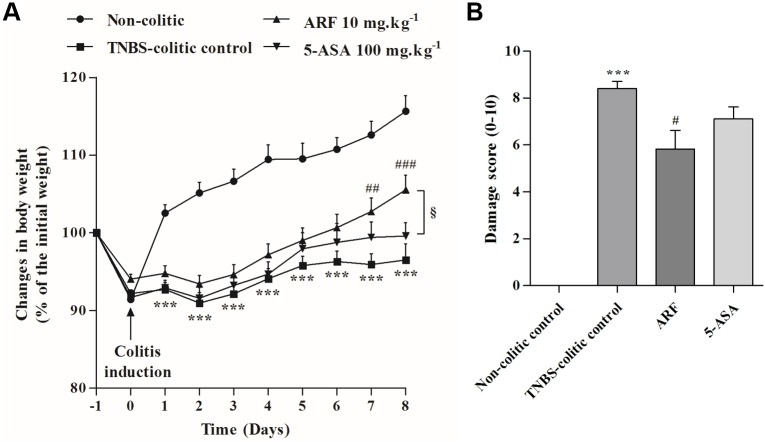
Effect of anthocyanin-rich fraction, as compared with 5-ASA, on body weight and on macroscopic colonic damage in rats with TNBS-induced colitis. After colitis induction, each group of animals was treated daily for 8 days with either ARF or 5-ASA and their effects on body weight were recorded overtime and expressed in terms of % of the initial weight (**A)**. After colon collection (day 8 post TNBS-induced colitis), macroscopic colitis severity was blindly assessed and characterized in terms of damage score (**B**). Values are mean ± SEM of at least 8–9 animals per group. ***P<0.001 vs non-colitic control; ^#^P<0.05, ^##^P<0.01, ^###^P<0.001 vs TNBS-colitic control; ^§^P<0.05 vs 5-ASA group.

During the study, the animals were constantly monitored for variations in body weight and general health. The inflammatory status in the TNBS-colitis control group was associated with severe anorexia and diarrhea, when compared to non-colitic control. Accordingly, as shown in **Fig 2A**, TNBS-colitis control group showed no significant weight gain with time, in contrast with the weight gain observed in healthy rats (115.7 ± 2.0%). Rats treated with ARF exhibited a significant recovery along the time, when compared with TNBS-colitis control group and the extent of recovery was clearly and statistically higher than that provided by 5-ASA, especially in the last two days. Notice that the observed benefits occurred at a concentration of anthocyanins about 30 times lower than that of 5-ASA (10 mg.kg^-1^ of ARF corresponds to 0.02 mmol.kg^-1^ in terms of malvidin-3-glucoside, while 100 mg.kg^-1^ of 5-ASA corresponds to 0.65 mmol.kg^-1^).

By the end of the experiment, the mortality in the TNBS-colitis control group was found to be very small (1/10), compared with the healthy, non-colitic control (0/10), indicating that the induced colonic injury was not fatal within 8 days, and neither the ARF nor 5-ASA, in the doses used, increased it. Additionally, neither TNBS nor the different treatments caused significant changes in the relative weights of the main organs (% of total body weight), such as liver, kidneys and heart (**S1 Table**) which are highly susceptible to drugs toxicity.

**Fig 3:**
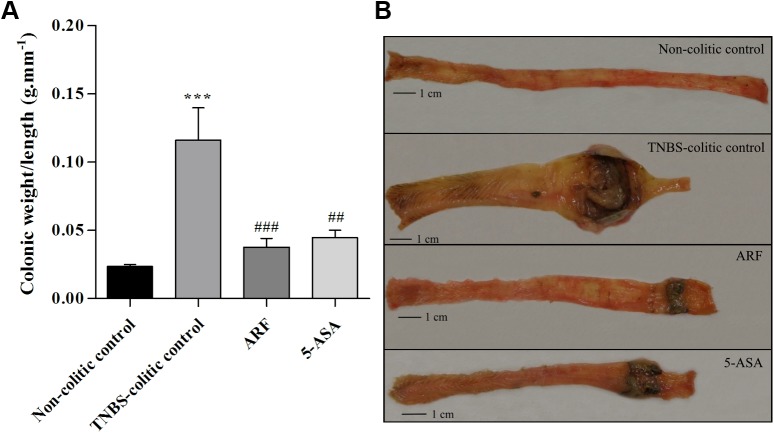
Effect of anthocyanin-rich fraction, as compared with 5-ASA, on the colon weight/length ratio of TNBS-induced colitic rats. On day 8, post TNBS-induced colitis, the colon of each animal was excised and evaluated in terms of weight and length. The colon weight vs length ratio for each group of animals is represented in graph **A**. Representative images of typical colons of each group are also shown (**B**). Values are mean ± SEM of at least 8–9 animals per group. ***P<0.001 vs non-colitic control; ^##^P<0.01, ^###^P<0.001 vs TNBS-colitic control.

The intracolonic administration of TNBS in ethanol induced colonic inflammation which was eight days later characterized by several events, namely: i) severe necrosis of the mucosa, typically extending 4–6 cm along the colon; ii) bowel wall thickening; iii) colon shortening; iv) hyperemia; and v) focal adhesions to adjacent organs, according to the scale represented in **Table 1**. Altogether, these data were correlated for each group of animals and shown in **Fig 2B**, indicating that the treatment with ARF counteracted the TNBS-induced damage score in a more efficient way than with 5-ASA. ARF also led to a very strong decrease in the colonic weight/length ratio (**Fig 3A**), being even more effective than 5-ASA, although in a much lower molar concentration than this drug. Representative colon pictures of each group are shown in **Fig 3B**.

### The anthocyanin-rich fraction inhibited the amount of active myeloperoxidase and the alkaline phosphatase activity, more efficiently than 5-ASA

**Fig 4:**
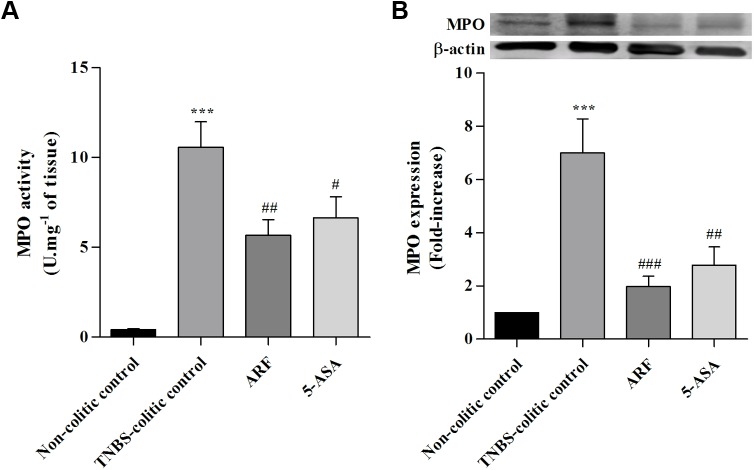
Effect of anthocyanin extract administration on colon myeloperoxidase activity and expression as compared with 5-ASA, in TNBS-induced colitis. MPO activity (**A**) was measured in colon tissue homogenates and expressed in units of enzyme activity per mg of tissue. MPO protein expression (**B**) was assessed in colon tissue samples by Western blotting and expressed as fold increase relative to the non-colitic control group. Values are mean ± SEM from at least 8–9 animals per group, each one in duplicate. ***P<0.001 vs non-colitic control; ^#^P<0.05, ^##^P<0.01, ^###^P<0.001 vs TNBS-colitic control.

The beneficial effects of our anthocyanin extract on TNBS-induced rat colitis were also studied biochemically in terms of well-established inflammatory markers. For this purpose, we analysed the MPO activity and its protein expression in colon sections from the different groups of rats, by the end of the experiment. The activity of this enzyme has been widely accepted as a strong marker of the inflammation degree, in terms of leukocyte infiltration in tissues [31]. As shown in **Fig 4**, TNBS led to a significant increase in either MPO activity **(A)** or its protein expression **(B)**, when compared with the non-colitic control rats. Treatment with ARF, along 8 days after colitis induction, reduced significantly MPO activity (**Fig 4A**) and its protein expression (**Fig 4B**) in a higher extent than with 5-ASA, indicating a higher anti-inflammatory action.

**Fig 5:**
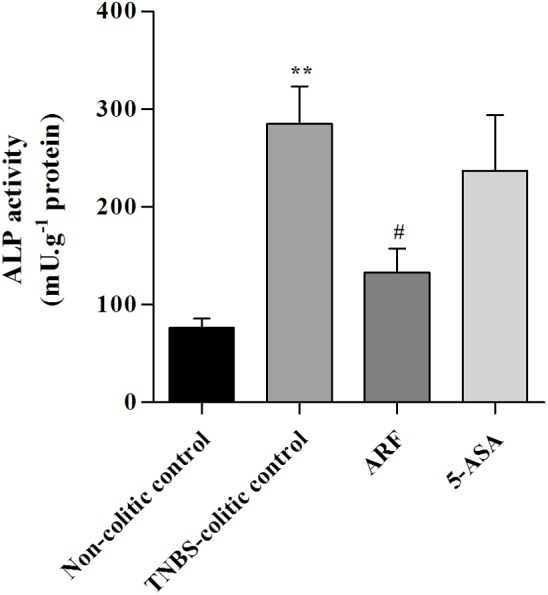
Effect of anthocyanin extract administration on colon alkaline phosphatase activity, as compared with 5-ASA, in TNBS-induced colitis. ALP activity was measured in colon tissue homogenates and expressed in units of enzyme activity per g of protein. Values are mean ± SEM from at least 8–9 animals per group, each one in duplicate. **P<0.01 vs non-colitic control; ^#^P<0.05, vs TNBS-colitic control.

Furthermore, we determined the ALP activity, since it has also been reported as a sensitive marker of inflammation in the intestine [33]. Results are shown in **Fig 5**and also indicated a significant capacity of anthocyanins to reduce this enzyme activity, in contrast with 5-ASA, whose effect was not statistically significant.

### The colonic antioxidant defenses were improved by the anthocyanin-rich fraction, more efficiently than by 5-ASA

**Fig 6:**
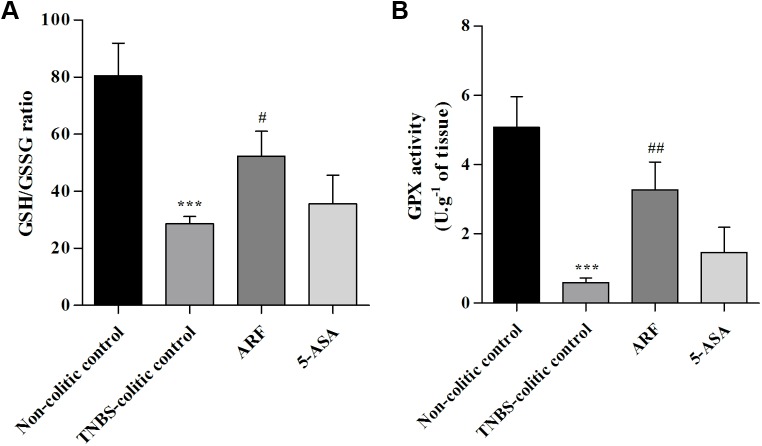
Effect of anthocyanin-rich fraction on colon GSH/GSSG ratio and GPX activity, as compared with 5-ASA, in TNBS-induced colitic rats. GSH/GSSG ratio and GPx activity were determined in samples of colonic tissue of each animal, as described in Materials and Methods. Statistics of GSH/GSSG ratio (**A**) and of GPx specific activity (**B**) for each group of animals are presented. Values are mean ± SEM obtained from at least 8–9 animals per group, each one in duplicate. ***P<0.001 vs non-colitic control; ^#^P<0.05, ^##^P<0.01 vs TNBS-colitic control.

An imbalance in the production of different reactive oxygen species, which may overwhelm the tissue antioxidant defenses, can be another molecular event involved in IBD [37]. Thus, to probe the colonic redox status, we decided to evaluate GSH content and GPx activity. In the TNBS-colitic control group, a great decrease (by about 65%) in the colonic GSH/GSSG ratio (**Fig 6A**), as well as in the GPx activity (by about 88%) (**Fig 6B**) was observed. ARF treatment significantly counteracted the induced decrease in GSH/GSSG ratio, indicating an improvement in the host antioxidant defenses, whereas 5-ASA was devoid of a significant effect, in the same experimental conditions (**Fig 6A**). Similar results were obtained for GPX activity (**Fig 6B**), showing a higher efficacy of anthocyanin fraction in recovering the antioxidant defenses, as compared with 5-ASA.

### The anthocyanin-rich fraction strongly counteracted TNBS-stimulated expression of colon COX-2 and iNOS enzymes

**Fig 7:**
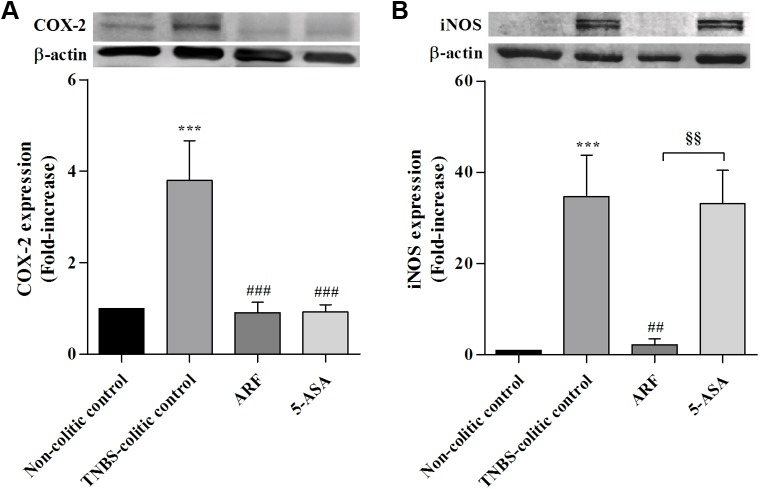
Comparison of the effects of anthocyanin-rich fraction and 5-ASA on COX-2 and iNOS protein expressions in colon tissue samples of colitic rats. COX-2 (**A**) and iNOS (**B**) protein expressions were evaluated by Western blotting in each animal of each group and expressed as fold increase relative to the non-colitic control group. Values are means ± SEM from of at least 8–9 animals per group, each one in duplicate. ***P<0.001 vs non-colitic control; ^##^P<0.01, ^###^P<0.001 vs TNBS-colitc control; ^§§^P<0.01 vs 5-ASA group.

The colonic inflammatory status was also characterized by a significant increase in the colonic COX-2 and iNOS protein expressions, relative to non-colitic control, as ascertained by Western blotting analysis from homogenates of inflamed tissue (**Fig 7**). Treatments with either ARF or 5-ASA strongly down-regulated COX-2 expression (**Fig 7A**), reverting it to control levels. This indicates that COX-2 inhibition is a relevant action pathway for anthocyanins, like for 5-ASA. Concerning the effects on iNOS expression, only the treatment with ARF could counteract drastically its increase in TNBS-induced colitis (by about 95%) whereas 5-ASA, in the dose used, did not show a significant effect (**Fig 7B**).

## Discussion

The present study was undertaken mainly to evaluate the potential use of an anthocyanin-rich fraction obtained from Portuguese blueberry fruits for the treatment of intestinal inflammation, in a TNBS induced colitis rat model, in comparison with 5-ASA, the standard therapy for most IBD patients. Our data confirmed the anti-inflammatory action previously reported to blueberry anthocyanins [14,20] and showed, for the first time, that the protection afforded by this blueberry anthocyanin mixture in a colitis rat model was much more effective than that provided by 5-ASA, in similar experimental conditions.

The ARF used presented a high content and great variety of anthocyanins, which is in agreement with previously reported findings from different cultivars around the world [26,38,39]. However, the anthocyanin profile was somewhat different, with malvidin as the major class, instead of cyanidin, and malvidin-3-galactoside and petunidin-3-arabinoside in the highest concentrations (Fig 1). It is worthy of note that these two anthocyanins have been detected in higher amounts than others in the ileostomy fluids of test patients after blueberries consumption [40], suggesting their potential relevance in modulating the intestinal inflammatory process.

The results obtained in this rat model of colitis showed that either ARF (10 mg.kg^-1^) or 5-ASA (100 mg.Kg^-1^) treatments counteracted the various signs of intestinal inflammation, including the very low rate of body weight gain, the colonic macroscopic damage score and the colon shortening, caused by TNBS (Figs 2 and 3). Nonetheless, the ARF treatment showed better improvements than 5-ASA, despite the much lower molar dose of anthocyanins used, indicating its higher effectiveness.

The intestinal anti-inflammatory potential of the anthocyanin-rich fraction was also demonstrated by the reduction of active MPO and by the inhibition of ALP activity. MPO is abundantly expressed in primary granules of neutrophils, thus, tissue-associated MPO activity and expression can be used as a fundamental and reliable biochemical marker of leukocyte infiltration and of tissue recovery [41]. In agreement with the macroscopic results previously referred, ARF treatment significantly reduced, and more efficiently than 5-ASA, MPO activity and its expression stimulated by TNBS-induced colitis (Fig 4A and 4B). Similarly, in what concerns intestinal ALP activity, ARF treatment demonstrated a better efficacy than 5-ASA in reducing this TNBS-induced enzyme activity (Fig 5). Currently, it is generally agreed that a tissue-nonspecific alkaline phosphatase isoform is essentially expressed in the colon and is upregulated in response to inflammation [33,42]. Also, in the inflamed colon, both colonocytes and infiltrating leukocytes account for the increased ALP activity. Moreover, it is known that this ALP isoform is specifically upregulated by oxidative stimulus [43]. Thus, since inflammation has been extensively associated with an increase in oxidative stress [37], the protection afforded by either anthocyanins or 5-ASA could be related to their well-known antioxidant properties [11,12,26,44].

On the other hand, TNBS-induced colitis has been associated with changes in antioxidant enzymes activity and in the levels of GSH [45], the major intracellular redox buffer, and decreases in GSH/GSSG ratio have been correlated with the severity of mucosal inflammation in IBD [46]. Our data showed a strong reduction in the intestinal GSH/GSSG ratio in the TNBS-induced colitis group, by about 65% (Fig 6A), indicating a severe decrease in intestinal redox status. In agreement with this, the intestinal GPX activity decreased drastically in the same group, by about 90% (Fig 6B). The relevance of this enzyme in the cellular antioxidant defense machinery is well known, by reducing hydroperoxides with the parallel oxidation of GSH to GSSG [16]. In this regard, the ARF treatment improved significantly either the GSH/GSSG ratio or the GPX activity in colon tissue, counteracting the TNBS-induced effects, in contrast with 5-ASA that did not significantly improve these oxidative stress markers (Fig 6A and 6B). Thus, the higher beneficial effect of anthocyanins in alleviating the severity of TNBS-induced colitis may by related, in part, with the higher ability to strengthen colon antioxidant defenses.

Besides the increase in oxidative stress, the excessive production of pro-inflammatory mediators is a key player to the intestinal damage progression in IBD. Concerning such mediators, both iNOS and COX-2 enzymes seem to play a synergistic role in inflammation onset and severity [2]. Upregulation of these enzymes expression is known to be induced by activation of different signaling pathways, namely the nuclear factor κB (NF-κB) and the signal transducer and activator of transcription 1 (STAT1), whose expressions and activities have been reported to increase during intestinal inflammation [13,47,48]. This work demonstrated that the treatment with anthocyanins could counteract drastically the strong overexpression of both iNOS and COX-2 enzymes induced by TNBS, while 5-ASA only counteracted COX-2 overexpression. Actually, the strong inhibition of COX-2 expression seems to be a crucial common mechanism underlying the anti-inflammatory actions of 5-ASA and ARF (Fig 7A). This observation is highly relevant given that this inducible form of COX is known to be upregulated in the inflamed gut of IBD patients [49,50], being a target for several drugs used in IBD, including 5-ASA [51]. Regarding the effects on iNOS overexpression, the results obtained reinforced the much higher anti-inflammatory capacity of anthocyanins when compared to 5-ASA, as they inhibited almost completely the enzyme expression, in contrast with 5-ASA which did not show any significant effect (Fig 7B) despite being used in a much higher molar dose than that of anthocyanins.

**In conclusion**, our data show for the first time the stronger anti-inflammatory activity of an anthocyanin-rich fraction obtained from the blueberry *Vaccinium corymbosum* L., grown in Portugal, on TNBS-induced rat colitis, in comparison with 5-ASA, a reference anti-inflammatory drug in IBD. Such activity is complex, involving different mechanisms including decrease in leukocyte infiltration, increase in antioxidant defenses and downregulation of proinflammatory enzymes. The strong inhibition of colon COX-2 expression seems to be a crucial anti-inflammatory mechanism common to 5-ASA and ARF, but the additional higher ability of anthocyanins to downregulate iNOS and to decrease leukocytes infiltration and to increase antioxidant defenses in colon may account for the much higher anti-inflammatory action of ARF. Taken together, these results point to the efficacy of this anthocyanin mixture to counteract colitis severity and gather conditions to a faster recovery, being a contribution to the development of a promising natural therapeutic approach for intestinal inflammation.

## Supporting information

S1 Table Statistics of liver, kidneys, and heart *vs* body weight (% of total body weight) for each group of animals. P-values were calculated using t-test analysis. P^(*)^>0.05, vs non-colitic control; P^(#)^>0.05, vs TNBS-colitic control; Thus, no statistical significance was observed.
(TIF)

## References

[1] Loddo I, Romano C (2015) Inflammatory bowel disease: genetics, epigenetics, and pathogenesis. Front Immunol 6: 551 10.3389/fimmu.2015.00551 26579126 PMC4629465

[2] Algieri F, Zorrilla P, Rodriguez-Nogales A, Garrido-Mesa N, Banuelos O, et al (2013) Intestinal anti-inflammatory activity of hydroalcoholic extracts of Phlomis purpurea L. and Phlomis lychnitis L. in the trinitrobenzenesulphonic acid model of rat colitis. J Ethnopharmacol 146: 750–759. 10.1016/j.jep.2013.01.041 23395625

[3] Lichtenstein GR, Hanauer SB, Sandborn WJ (2009) Management of Crohn's disease in adults. Am J Gastroenterol 104: 465–483; quiz 464, 484. 10.1038/ajg.2008.168 19174807

[4] Serra D, Rufino AT, Mendes AF, Almeida LM, Dinis TC (2014) Resveratrol modulates cytokine-induced Jak/STAT activation more efficiently than 5-aminosalicylic acid: an in vitro approach. PLoS One 9: e109048 10.1371/journal.pone.0109048 25271420 PMC4182878

[5] Liu L, Liu Z, Zhang T, Shi L, Zhang W, et al (2015) Combined therapy with Rheum tanguticum polysaccharide and low-dose 5-ASA ameliorates TNBS-induced colitis in rats by suppression of NF-kappaB. Planta Med 81: 705–712. 10.1055/s-0035-1545945 26069953

[6] Farzaei MH, Rahimi R, Abdollahi M (2015) The role of dietary polyphenols in the management of inflammatory bowel disease. Curr Pharm Biotechnol 16: 196–210. 25601607 10.2174/1389201016666150118131704

[7] He J, Giusti MM (2010) Anthocyanins: natural colorants with health-promoting properties. Annu Rev Food Sci Technol 1: 163–187. 10.1146/annurev.food.080708.100754 22129334

[8] Lee SG, Kim B, Yang Y, Pham TX, Park YK, et al (2014) Berry anthocyanins suppress the expression and secretion of proinflammatory mediators in macrophages by inhibiting nuclear translocation of NF-kappaB independent of NRF2-mediated mechanism. J Nutr Biochem 25: 404–411. 10.1016/j.jnutbio.2013.12.001 24565673

[9] Sodagari HR, Farzaei MH, Bahramsoltani R, Abdolghaffari AH, Mahmoudi M, et al (2015) Dietary anthocyanins as a complementary medicinal approach for management of inflammatory bowel disease. Expert Rev Gastroenterol Hepatol: 1–14. 10.1586/17474124.2015.1002086 25586636

[10] Esposito D, Chen A, Grace MH, Komarnytsky S, Lila MA (2014) Inhibitory effects of wild blueberry anthocyanins and other flavonoids on biomarkers of acute and chronic inflammation in vitro. J Agric Food Chem 62: 7022–7028. 10.1021/jf4051599 24397282

[11] Paixao J, Dinis TC, Almeida LM (2011) Dietary anthocyanins protect endothelial cells against peroxynitrite-induced mitochondrial apoptosis pathway and Bax nuclear translocation: an in vitro approach. Apoptosis 16: 976–989. 10.1007/s10495-011-0632-y 21785847

[12] Paixao J, Dinis TC, Almeida LM (2012) Malvidin-3-glucoside protects endothelial cells up-regulating endothelial NO synthase and inhibiting peroxynitrite-induced NF-kB activation. Chem Biol Interact 199: 192–200. 10.1016/j.cbi.2012.08.013 22959858

[13] Serra D, Paixão J, Nunes C, Dinis TCP, Almeida LM (2013) Cyanidin-3-glucoside suppresses cytokine-induced inflammatory response in human intestinal cells: comparison with 5-aminosalicylic acid. PLoS One 8: e73001 10.1371/journal.pone.0073001 24039842 PMC3765207

[14] Li L, Wang L, Wu Z, Yao L, Wu Y, et al (2014) Anthocyanin-rich fractions from red raspberries attenuate inflammation in both RAW264.7 macrophages and a mouse model of colitis. Sci Rep 4: 6234 10.1038/srep06234 25167935 PMC4148654

[15] Huang W, Zhu Y, Li C, Sui Z, Min W (2016) Effect of blueberry anthocyanins malvidin and glycosides on the antioxidant properties in endothelial cells. Oxid Med Cell Longev 2016: 1591803 10.1155/2016/1591803 27034731 PMC4789434

[16] Bhattacharyya A, Chattopadhyay R, Mitra S, Crowe SE (2014) Oxidative stress: an essential factor in the pathogenesis of gastrointestinal mucosal diseases. Physiol Rev 94: 329–354. 10.1152/physrev.00040.2012 24692350 PMC4044300

[17] Yi W, Akoh CC, Fischer J, Krewer G (2006) Absorption of anthocyanins from blueberry extracts by caco-2 human intestinal cell monolayers. J Agric Food Chem 54: 5651–5658. 10.1021/jf0531959 16848559

[18] McGhie TK, Walton MC (2007) The bioavailability and absorption of anthocyanins: towards a better understanding. Mol Nutr Food Res 51: 702–713. 10.1002/mnfr.200700092 17533653

[19] Romier B, Schneider YJ, Larondelle Y, During A (2009) Dietary polyphenols can modulate the intestinal inflammatory response. Nutr Rev 67: 363–378. 10.1111/j.1753-4887.2009.00210.x 19566597

[20] Wu LH, Xu ZL, Dong D, He SA, Yu H (2011) Protective effect of anthocyanins extract from blueberry on TNBS-induced IBD model of mice. Evid Based Complement Alternat Med 2011: 525462 10.1093/ecam/neq040 21785630 PMC3135784

[21] Oszmianski J, Ramos T, Bourzeix M (1988) Fractionation of phenolic compounds in red wine. Am J Enol Vitic 39: 259–262.

[22] Youdim KA, McDonald J, Kalt W, Joseph JA (2002) Potential role of dietary flavonoids in reducing microvascular endothelium vulnerability to oxidative and inflammatory insults small star, filled). J Nutr Biochem 13: 282–288. 12015158 10.1016/s0955-2863(01)00221-2

[23] Rodriguez-Saona LE, Wrolstad RE (2001) Extraction, isolation, and purification of anthocyanins. Current Protocols in Food Analytical Chemistry.F:F1:F1.1

[24] George S, Brat P, Alter P, Amiot MJ (2005) Rapid determination of polyphenols and vitamin C in plant-derived products. J Agric Food Chem 53: 1370–1373. 10.1021/jf048396b 15740008

[25] Giusti MM, Wrolstad RE (2001) Characterization and measurement of anthocyanins by UV-visible spectroscopy. Current Protocols in Food Analytical Chemistry. F:F1:F1.2.

[26] Faria A, Oliveira J, Neves P, Gameiro P, Santos-Buelga C, et al (2005) Antioxidant properties of prepared blueberry (Vaccinium myrtillus) extracts. J Agric Food Chem 53: 6896–6902. 10.1021/jf0511300 16104817

[27] Ancha HR, Kurella RR, McKimmey CC, Lightfoot S, Harty RF (2008) Luminal antioxidants enhance the effects of mesalamine in the treatment of chemically induced colitis in rats. Exp Biol Med (Maywood) 233: 1301–1308. 18703751 10.3181/0805-RM-140

[28] Reagan-Shaw S, Nihal M, Ahmad N (2008) Dose translation from animal to human studies revisited. FASEB J 22: 659–661. 10.1096/fj.07-9574LSF 17942826

[29] Siddiqui A, Ancha H, Tedesco D, Lightfoot S, Stewart CA, et al (2006) Antioxidant therapy with N-acetylcysteine plus mesalamine accelerates mucosal healing in a rodent model of colitis. Dig Dis Sci 51: 698–705. 10.1007/s10620-006-3194-z 16614991

[30] Bell CJ, Gall DG, Wallace JL (1995) Disruption of colonic electrolyte transport in experimental colitis. Am J Physiol 268: G622–630. 7733288 10.1152/ajpgi.1995.268.4.G622

[31] Stucchi AF, Shofer S, Leeman S, Materne O, Beer E, et al (2000) NK-1 antagonist reduces colonic inflammation and oxidative stress in dextran sulfate-induced colitis in rats. Am J Physiol Gastrointest Liver Physiol 279: G1298–1306. 11093954 10.1152/ajpgi.2000.279.6.G1298

[32] Bessey OA, Lowry OH, Brock MJ (1946) A method for the rapid determination of alkaline phosphates with five cubic millimeters of serum. J Biol Chem 164: 321–329. 20989492

[33] Sanchez de Medina F, Martinez-Augustin O, Gonzalez R, Ballester I, Nieto A, et al (2004) Induction of alkaline phosphatase in the inflamed intestine: a novel pharmacological target for inflammatory bowel disease. Biochem Pharmacol 68: 2317–2326. 10.1016/j.bcp.2004.07.045 15548378

[34] Griffith OW (1980) Determination of glutathione and glutathione disulfide using glutathione reductase and 2-vinylpyridine. Anal Biochem 106: 207–212. 7416462 10.1016/0003-2697(80)90139-6

[35] Rahman I, Kode A, Biswas SK (2006) Assay for quantitative determination of glutathione and glutathione disulfide levels using enzymatic recycling method. Nat Protoc 1: 3159–3165. 10.1038/nprot.2006.378 17406579

[36] Sanchez-Fidalgo S, Cardeno A, Villegas I, Talero E, de la Lastra CA (2010) Dietary supplementation of resveratrol attenuates chronic colonic inflammation in mice. Eur J Pharmacol 633: 78–84. 10.1016/j.ejphar.2010.01.025 20132809

[37] Maor I, Rainis T, Lanir A, Lavy A (2008) Oxidative stress, inflammation and neutrophil superoxide release in patients with Crohn's disease: distinction between active and non-active disease. Dig Dis Sci 53: 2208–2214. 10.1007/s10620-007-0141-6 18253831

[38] Scalzo J, Stevenson D, Hedderley D (2013) Blueberry estimated harvest from seven new cultivars: fruit and anthocyanins. Food Chem 139: 44–50. 10.1016/j.foodchem.2013.01.091 23561076

[39] Bunea A, Rugina D, Sconta Z, Pop RM, Pintea A, et al (2013) Anthocyanin determination in blueberry extracts from various cultivars and their antiproliferative and apoptotic properties in B16-F10 metastatic murine melanoma cells. Phytochemistry 95: 436–444. 10.1016/j.phytochem.2013.06.018 23890760

[40] Kahle K, Kraus M, Scheppach W, Ackermann M, Ridder F, et al (2006) Studies on apple and blueberry fruit constituents: do the polyphenols reach the colon after ingestion? Mol Nutr Food Res 50: 418–423. 10.1002/mnfr.200500211 16548015

[41] Rosillo MA, Sanchez-Hidalgo M, Cardeno A, Aparicio-Soto M, Sanchez-Fidalgo S, et al (2012) Dietary supplementation of an ellagic acid-enriched pomegranate extract attenuates chronic colonic inflammation in rats. Pharmacol Res 66: 235–242. 10.1016/j.phrs.2012.05.006 22677088

[42] Dahan S, Roth-Walter F, Arnaboldi P, Agarwal S, Mayer L (2007) Epithelia: lymphocyte interactions in the gut. Immunol Rev 215: 243–253. 10.1111/j.1600-065X.2006.00484.x 17291293 PMC3864677

[43] Lalles JP (2014) Intestinal alkaline phosphatase: novel functions and protective effects. Nutr Rev 72: 82–94. 10.1111/nure.12082 24506153

[44] Dinis TC, Maderia VM, Almeida LM (1994) Action of phenolic derivatives (acetaminophen, salicylate, and 5-aminosalicylate) as inhibitors of membrane lipid peroxidation and as peroxyl radical scavengers. Arch Biochem Biophys 315: 161–169. 7979394 10.1006/abbi.1994.1485

[45] Nieto N, Torres MI, Fernandez MI, Giron MD, Rios A, et al (2000) Experimental ulcerative colitis impairs antioxidant defense system in rat intestine. Dig Dis Sci 45: 1820–1827. 11052326 10.1023/a:1005565708038

[46] Circu ML, Aw TY (2012) Intestinal redox biology and oxidative stress. Semin Cell Dev Biol 23: 729–737. 10.1016/j.semcdb.2012.03.014 22484611 PMC3396776

[47] Atreya I, Atreya R, Neurath MF (2008) NF-kappaB in inflammatory bowel disease. J Intern Med 263: 591–596. 10.1111/j.1365-2796.2008.01953.x 18479258

[48] Schreiber S, Rosenstiel P, Hampe J, Nikolaus S, Groessner B, et al (2002) Activation of signal transducer and activator of transcription (STAT) 1 in human chronic inflammatory bowel disease. Gut 51: 379–385. 12171960 10.1136/gut.51.3.379 PMC1773344

[49] Singer II, Kawka DW, Schloemann S, Tessner T, Riehl T, et al (1998) Cyclooxygenase 2 is induced in colonic epithelial cells in inflammatory bowel disease. Gastroenterology 115: 297–306. 9679035 10.1016/s0016-5085(98)70196-9

[50] Chun KS, Surh YJ (2004) Signal transduction pathways regulating cyclooxygenase-2 expression: potential molecular targets for chemoprevention. Biochem Pharmacol 68: 1089–1100. 10.1016/j.bcp.2004.05.031 15313405

[51] Miyoshi J, Yajima T, Shimamura K, Matsuoka K, Okamoto S, et al (2012) 5-aminosalicylic acid mediates expression of cyclooxygenase-2 and 15-hydroxyprostaglandin dehydrogenase to suppress colorectal tumorigenesis. Anticancer Res 32: 1193–1202. 22493349

