# Supplementary figures and images for: Comparison of anti-inflammatory activities of an anthocyanin-rich fraction from Portuguese blueberries (Vaccinium corymbosum L.) and 5-aminosalicylic acid in a TNBS-induced colitis rat model
Source: PLoS One. 2017 Mar 22;12(3):e0174116. doi: 10.1371/journal.pone.0174116 (PMC5362129; doi:10.1371/journal.pone.0174116)

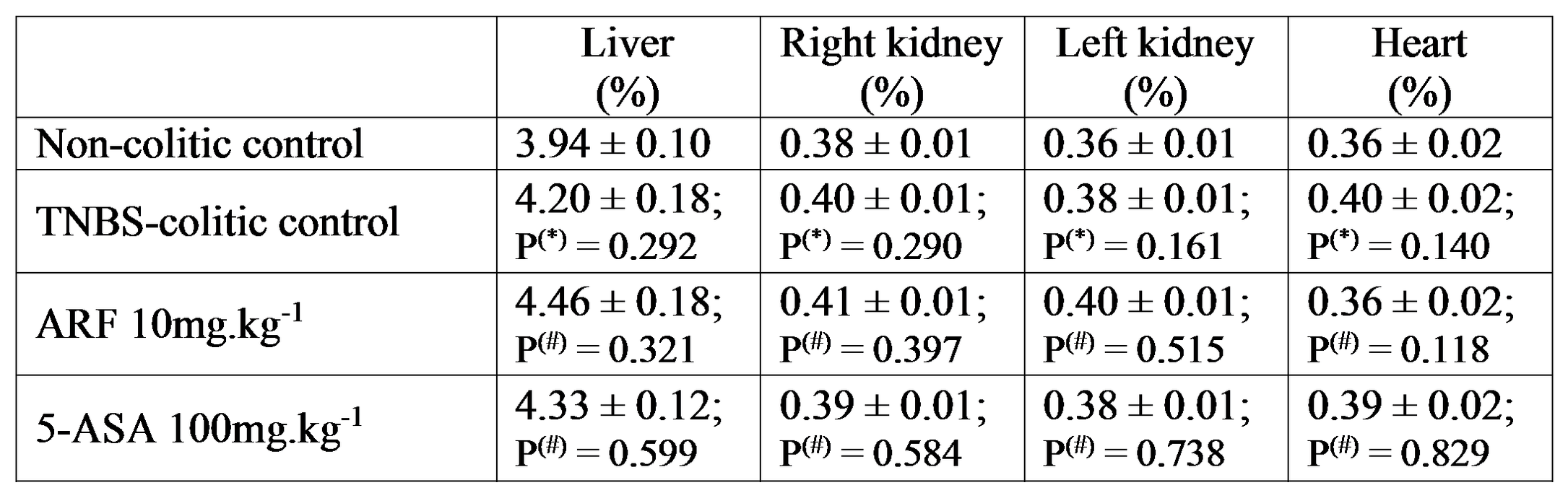

Supplement: S1 Table — P-values were calculated using t-test analysis. P(*)>0.05, vs non-colitic control; P(#)>0.05, vs TNBS-colitic control; Thus, no statistical significance was observed. (TIF) [file pone.0174116.s001.tif]
